# Supplementary material for: L-SCRaMbLE as a tool for light-controlled Cre-mediated recombination in yeast
Source: Nat Commun. 2018 May 22;9:1931. doi: 10.1038/s41467-017-02208-6 (PMC5964156; doi:10.1038/s41467-017-02208-6)
Supplement: Supplementary file 1 — Supplementary Information [file 41467_2017_2208_MOESM1_ESM.pdf]

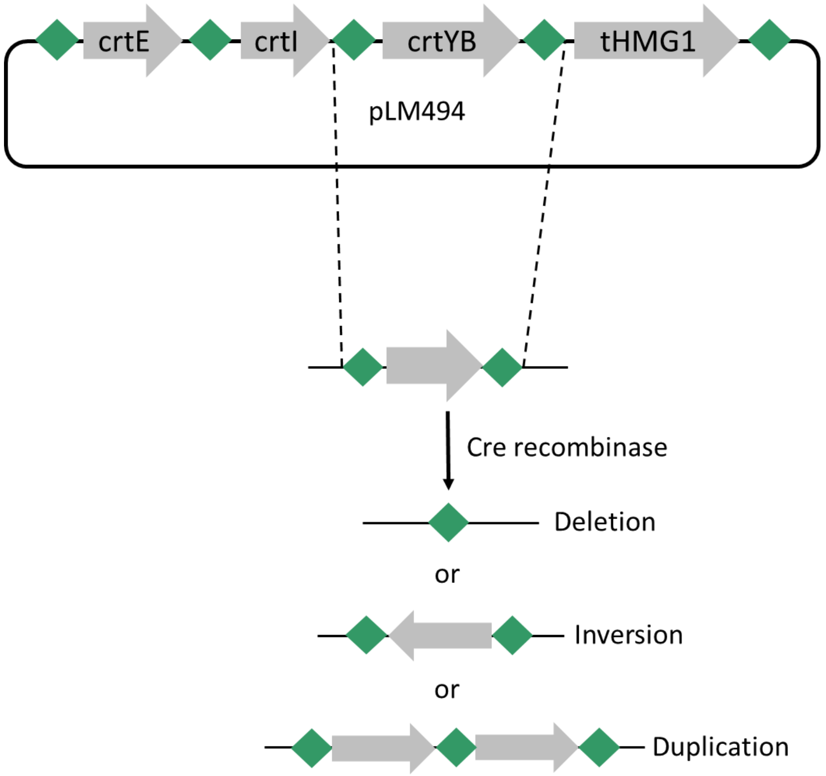

Supplementary Figure 1: Schematic presentation of plasmid pLM494 and possible recombination outcomes. Plasmid pLM494 carries four genes of the β-carotene pathway and each gene is flanked by *loxPsym* sites. Cre recombinase activity can cause deletion, inversion or duplication of each *loxPsym*-flanked gene.

A 4 h

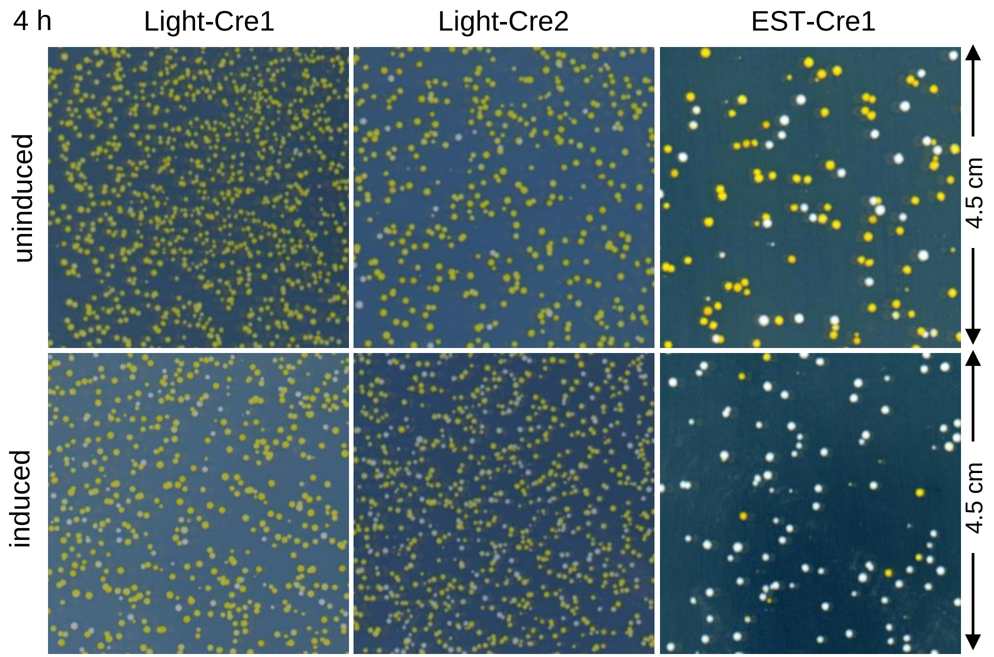

	uninduced		induced	
	White colonies	Counted colonies	White colonies	Counted colonies
Light-Cre1	2.0% ± 1.6	~160	14.0% ± 0.8	~250
Light-Cre2	10.0% ± 3.7	~110	27.0% ± 2.9	~160
EST-Cre1	40.0% ± 5.9	~120	94.0% ± 2.2	~215

B 16 h

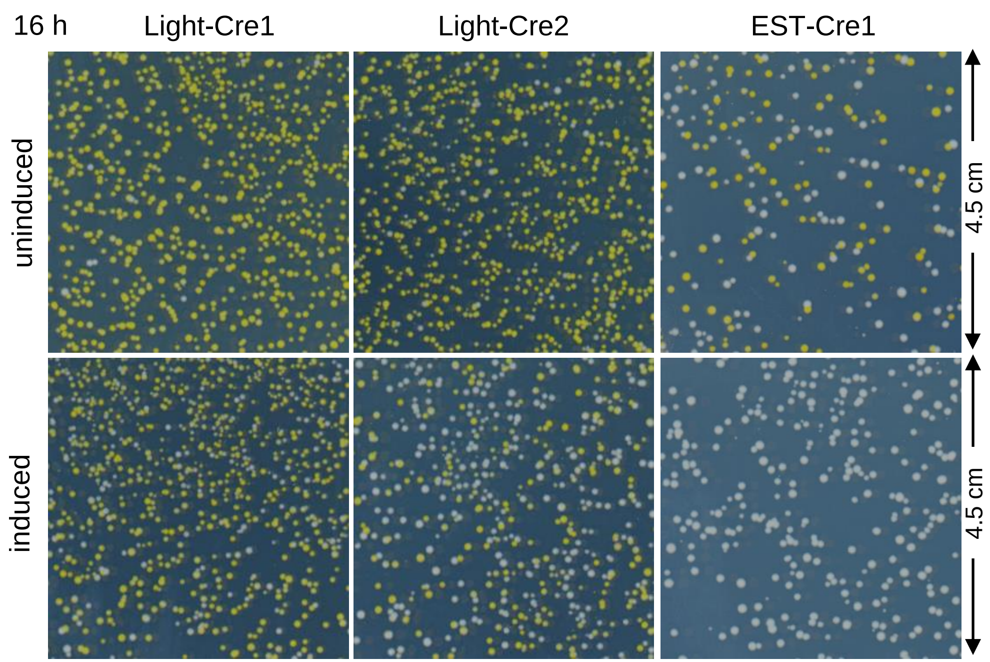

	uninduced		induced	
	White colonies	Counted colonies	White colonies	Counted colonies
Light-Cre1	1.3% ± 0.5	~225	32.3% ± 1.3	~215
Light-Cre2	6.7% ± 2.6	~210	46.7% ± 5.3	~210
EST-Cre1	45.0% ± 7.8	~230	100.0% ± 0	~225

C 24 h

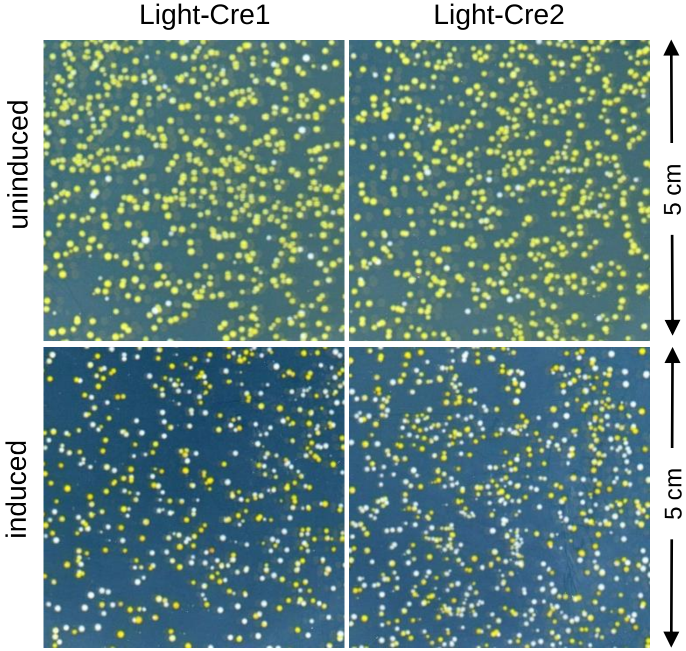

	uninduced		induced	
	White colonies	Counted colonies	White colonies	Counted colonies
Light-Cre1	5.2% ± 2.6	~220	52.0% ± 1.8	~260
Light-Cre2	8.1% ± 6.2	~260	52.5% ± 8.0	~240

Supplementary Figure 2: Recombination experiment with incubation times of 4 h, 16 h and 24 h. Light-Cre1, Light-Cre2 and EST-Cre1 cells were grown for 6 h in darkness. After induction with a 5-min red light pulse (Light-Cre1/2) or with 1 µM β-estradiol (EST-Cre1), samples were grown for further **A** 4 h, **B** 16 h, and **C** 24 h at 30°C and 230 rpm with 10-sec red light pulses every 5 min or in the dark. One ml of each culture was pelleted, diluted, plated on appropriate SC drop-out medium, and grown for another 2 d at 30°C in the dark. Each experiment was performed in three independent replicates. Plates show the result of one representative replicate of each strain. The tables below the figures show values for the counted colonies (first row) and the percentage of white colonies (second row) for all three replicates.

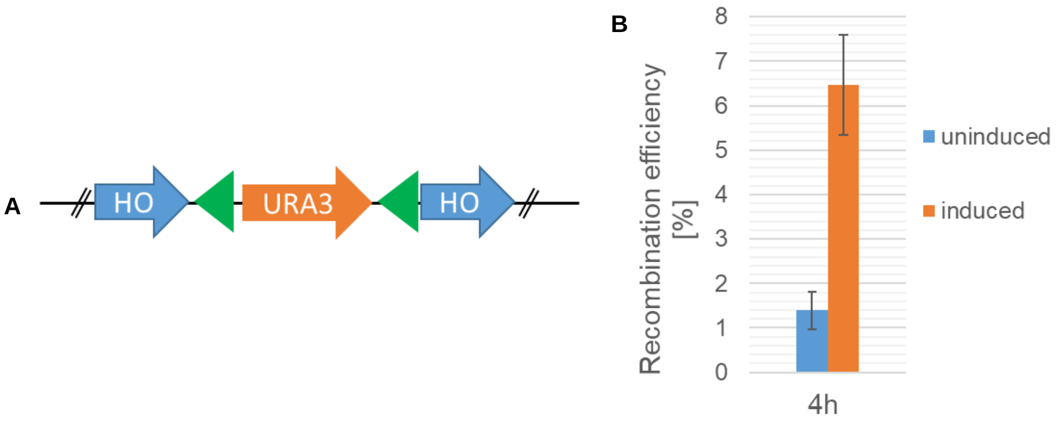

C

Construct	Medium conditions	Induced samples		Uninduced samples	
		Colony number	Recombination efficiency [%]	Colony number	Recombination efficiency [%]
EST-Cre2_1	SC-His+FOA	73	8.0	13	1.2
	SC-His	908		1032	
EST-Cre2_2	SC-His+FOA	60	5.9	19	2.0
	SC-His	1020		969	
EST-Cre2_3	SC-His+FOA	41	5.5	8	1.0
	SC-His	748		832	

Supplementary Figure 3: Testing pLM006 with a genome-integrated *loxP*-flanked *URA3* cassette. A A *loxP-URA3-loxP* cassette was integrated into the *HO* locus of the *S. cerevisiae* genome resulting in strain yLM1295. Plasmid pLM006 was transformed into yLM1295 to generate strain EST-Cre2. Beta-estradiol induction will cause recombination of *loxP* sites and subsequently deletion of the *URA3* cassette. When plating cells on medium containing FOA, cells expressing *URA3* will die, while cells lacking the *URA3* expression cassette can grow on FOA containing medium. **B** EST-Cre2 cells were grown for 6 h in darkness. After induction with 1 µM β-estradiol, samples were grown for further 4 h at 30°C and 230 rpm. Hundred µl of each culture was pelleted, diluted, plated on appropriate SC drop-out medium, and grown for another 2 d at 30°C. Each experiment was performed in three independent replicates. **C** The table shows values for the counted colonies and the percentage of recombination efficiency for all three replicates.

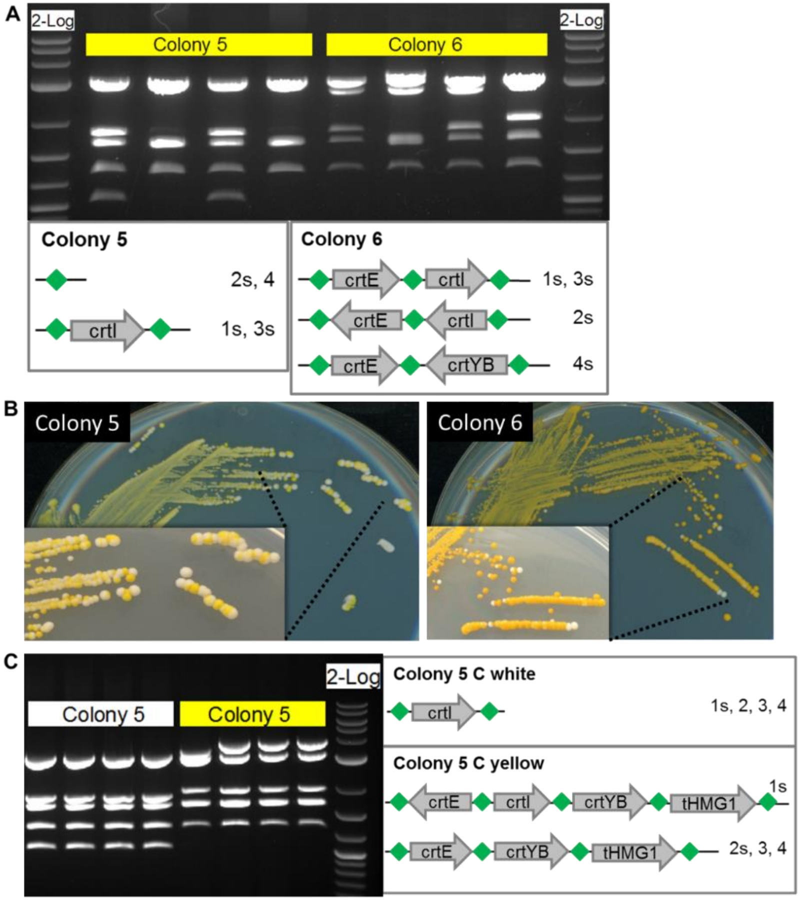

Supplementary Figure 4: Reanalysis of colonies 5 and 6. A Parental colonies 5 and 6 were analyzed by isolating plasmids and subsequent transformation into *E. coli.* From each transformation, four individual colonies were chosen for plasmid isolation and restriction analysis with *Pst*I and *Sac*I. Below the gel picture, a schematic overview of the recombination events based on restriction pattern and sequencing results (indicated by 's') is shown. Numbers refer to the lanes on the gel picture. Green diamonds indicate *loxPsym* sites. **B** Parental colonies 5 and 6 were streaked out to obtain single colonies. **C** White and yellow sibling colonies from parental colony 5 (B, left panel) were analyzed by plasmid preparation and restriction digestion with *Pst*I and *Sac*I. Overview of recombination events is given as in **A**.

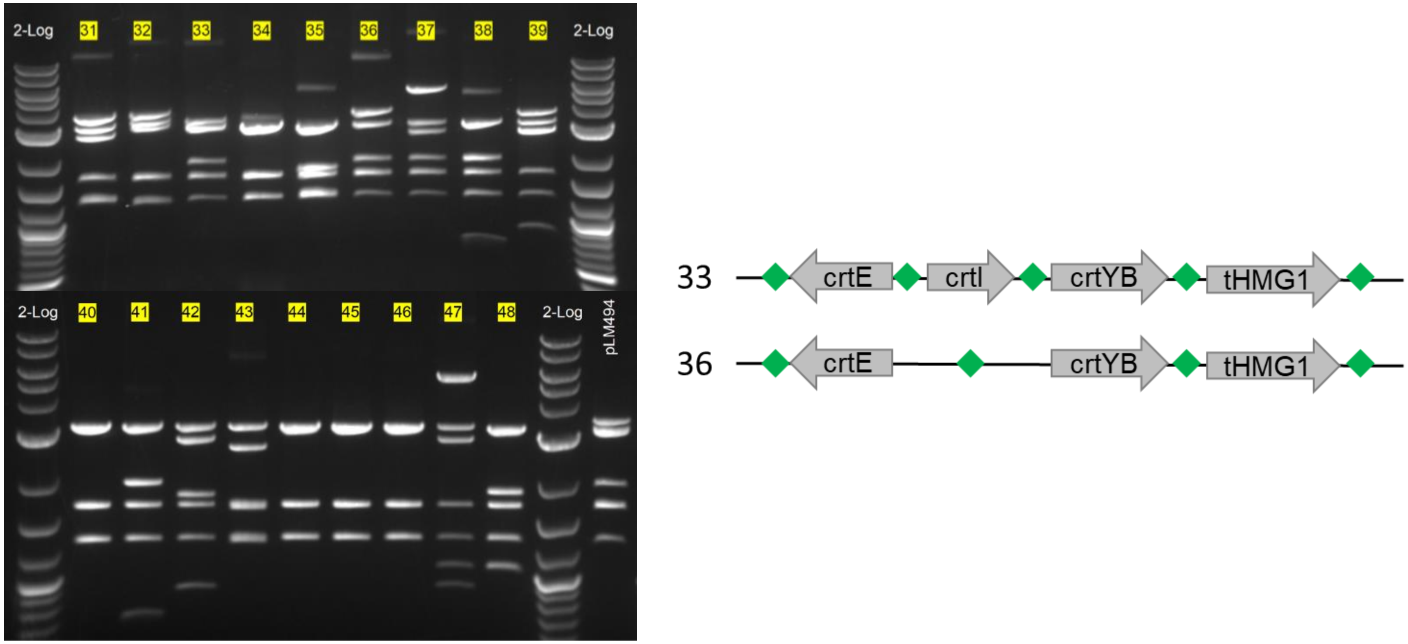

Supplementary Figure 5: Restriction patterns of pLM494 after Cre-mediated recombination. Plasmids were isolated from different yellow yeast colonies of induced Light-Cre1 cultures (16 h) and after passage through *E. coli* digested with *Pst*I and *Sac*I, and separated via gel electrophoresis. ´2-Log´, DNA Ladder (NEB). On the right recombination events of sequenced plasmids 33 and 36 are shown.

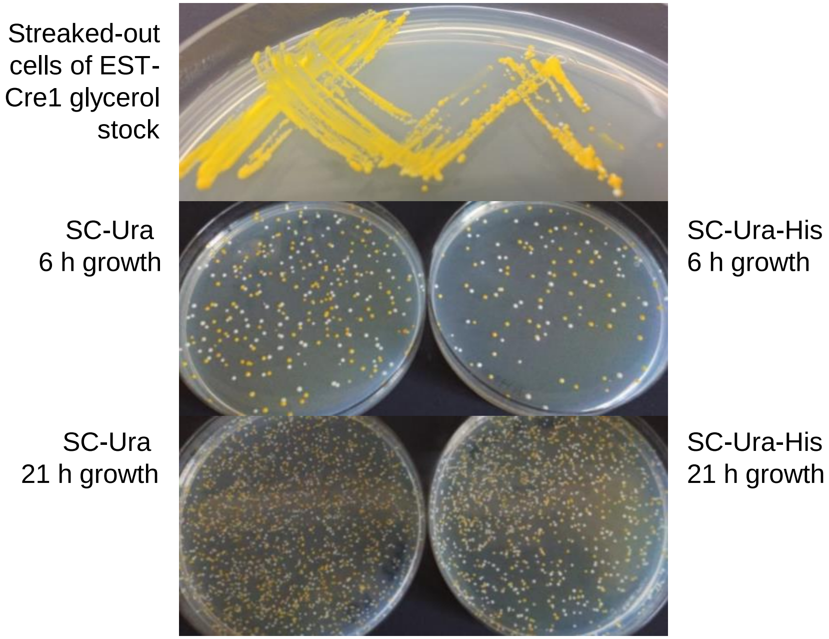

Supplementary Figure 6: Investigations of basal activity of EST-Cre1. A glycerol stock of yeast strain EST-Cre1 was streaked out on SC-Ura-His media. Overnight cultures were inoculated from four individual yellow colonies and grown for 6 h and 21 h, respectively. Cells (1 ml) were pelleted, resuspended in 1 ml ddH$_2$O, diluted 1:10 in ddH$_2$O, and 100 µl were plated on SC-Ura and SC-Ura-His plates.

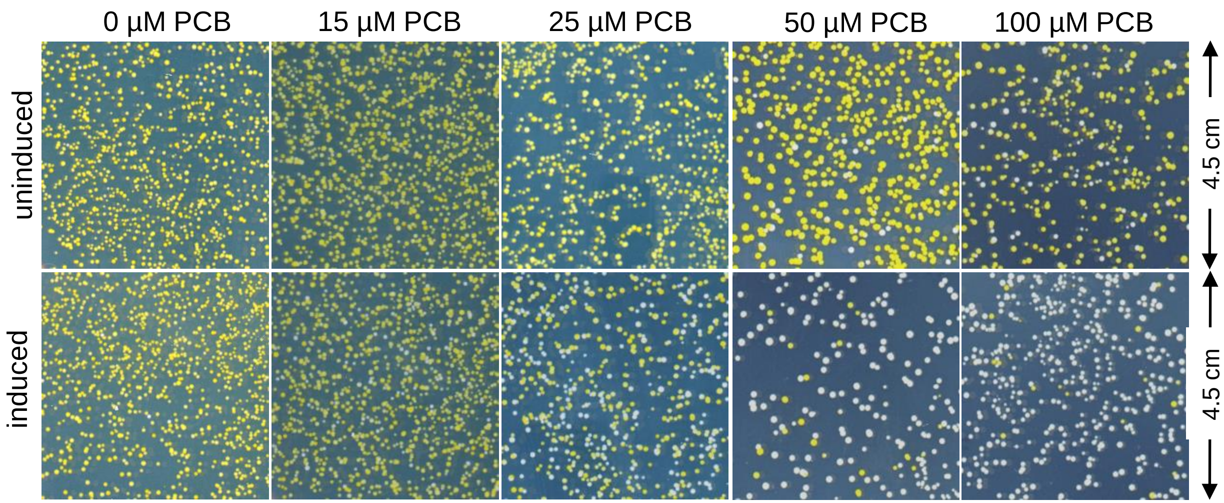

	Uninduced (% of white colonies)	Induced (% of white colonies)
0 µM PCB	0.5% ± 0.1	0.8% ± 0.3
15 µM PCB	0.6% ± 0.2	10.3% ± 1.7
25 µM PCB	0.7% ± 0.2	47.3% ± 7.1
50 µM PCB	3.4% ± 0.6	63.8% ± 4.5
100 µM PCB	10.0% ± 1.9	89.5% ± 5.1

Supplementary Figure 7: Recombination experiment with varying PCB concentrations. Light-Cre1 was grown in SC-Ura-Leu medium with 0, 15, 25, 50 or 100 µM PCB for 6 h in darkness after a far-red light pulse of 1 min. Thereafter, cells were induced with a 5-min red light pulse and grown for further 16 h with red light pulses of 10 sec, applied every 5 min at 30°C and 230 rpm. Uninduced cells were grown in the dark for 16 h. Hundred µl of each culture was pelleted, diluted, plated on appropriate SC drop-out medium, and grown for another 2 d. Each experiment was performed in three independent replicates. Around 400 colonies were counted for each plate. Plates show the results of one representative replicate with each PCB concentration. The table below shows values for the counted colonies (first row) and the percentage of white colonies (second row) for all three replicates.

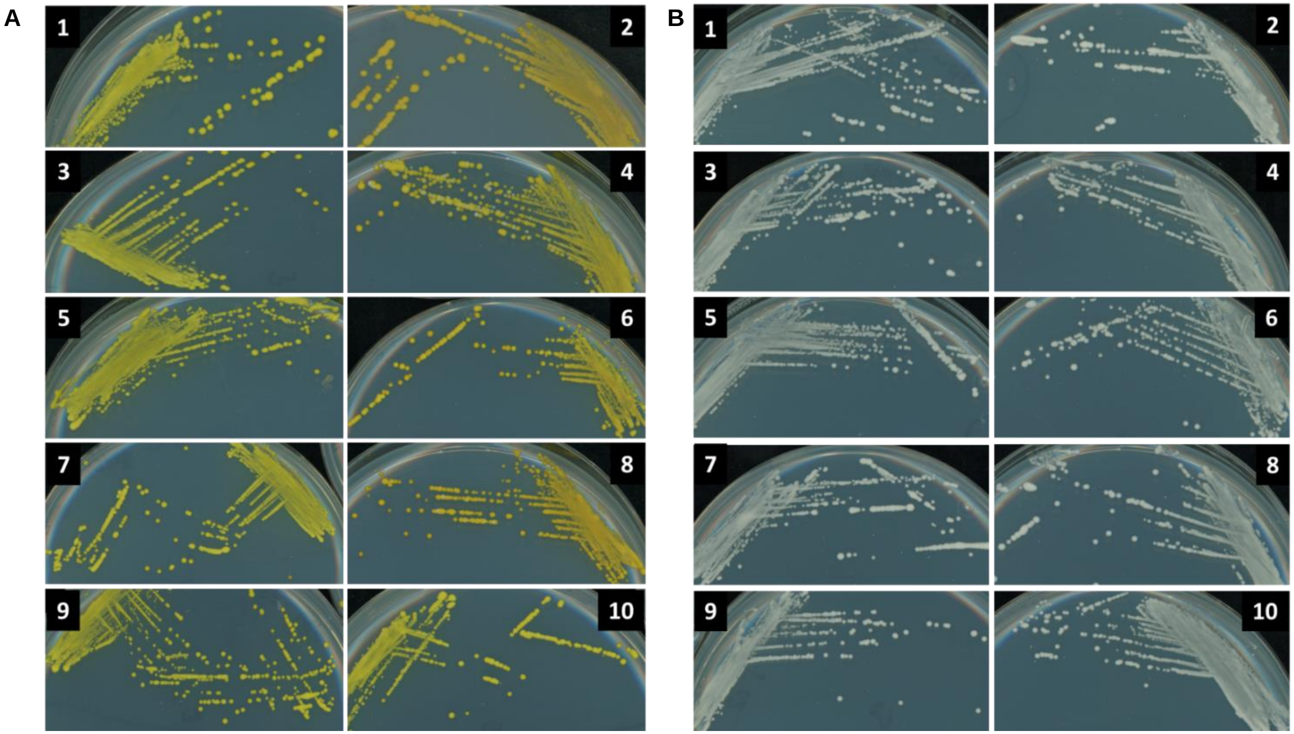
A
1
2
3
4
5
6
7
8
9
10
B
1
2
3
4
5
6
7
8
9
10

C

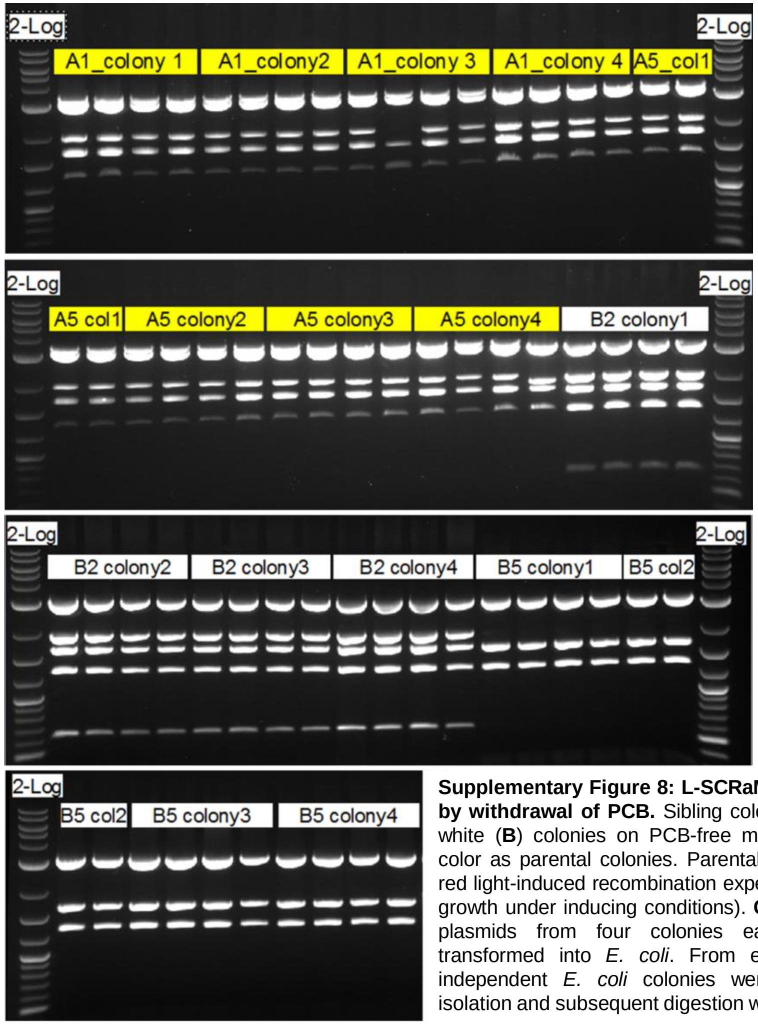

Supplementary Figure 8: L-SCRaMbLE can be inactivated by withdrawal of PCB. Sibling colonies from yellow (**A**) and white (**B**) colonies on PCB-free medium develop the same color as parental colonies. Parental colonies resulted from a red light-induced recombination experiment (25 µM PCB, 16 h growth under inducing conditions). **C** For A1, A5, B2 and B5, plasmids from four colonies each were isolated and transformed into *E. coli*. From each transformation, four independent *E. coli* colonies were analyzed by plasmid isolation and subsequent digestion with *Pst*I and *Sac*I.

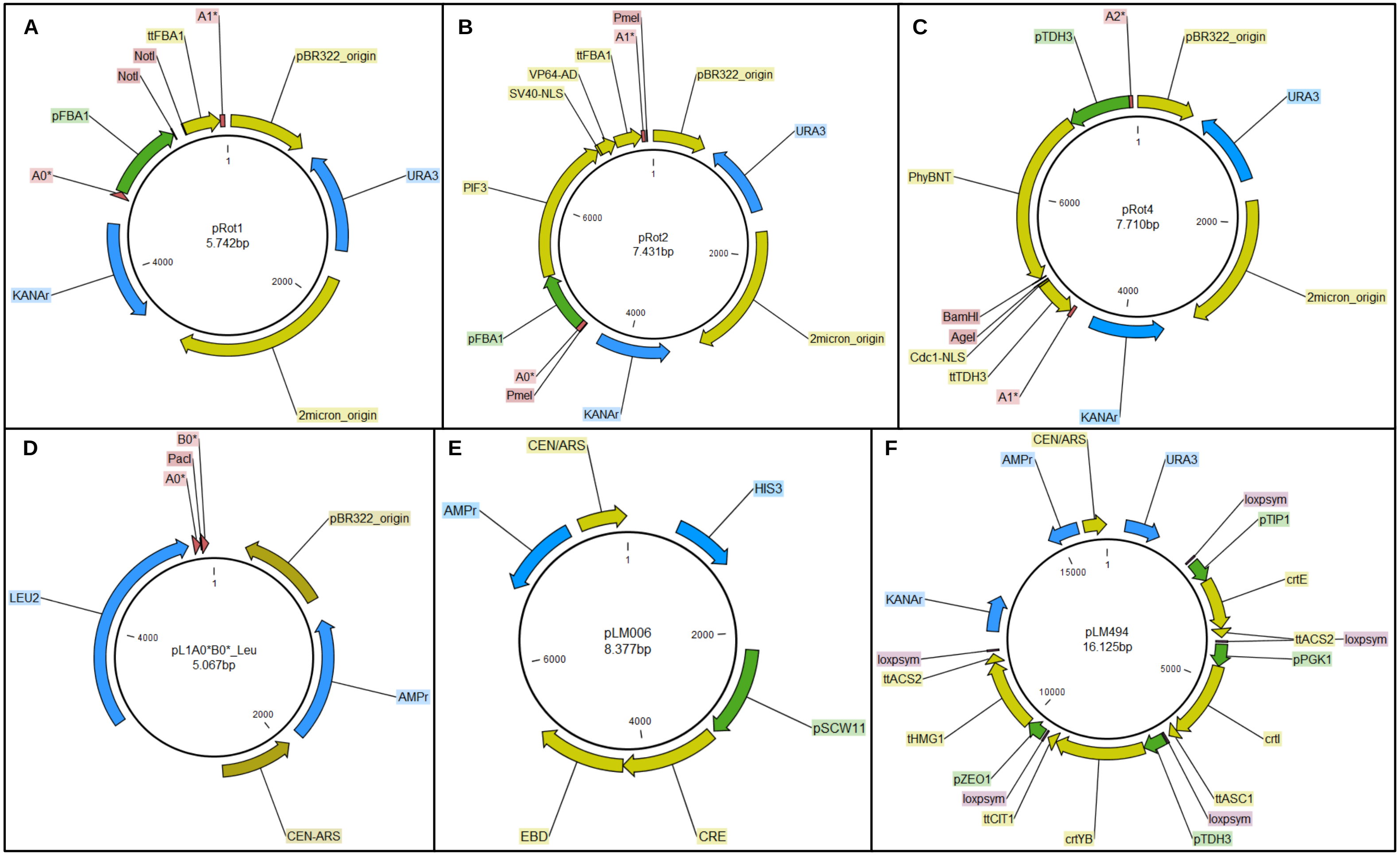

Supplementary Figure 9: Maps of plasmids pL1A0*B0*_Leu, pRot1, pRot2, pRot4, pLM006, and pLM494. Maps are shown for **A** Level 1 backbone pL1A0*B0*_Leu, precursor Level 0 vectors, **B** pRot1, **C** pRot2, **D** pRot4, **E** plasmid for β-estradiol inducible Cre pLM006, and **F** plasmid pLM494 harboring β-carotene biosynthesis genes, flanked with *loxPsym* sites. KANAr: kanamycin resistance, pFBA1: FBA1 promoter, ttFBA1: FBA1 terminator, PIF3: Phytochrome interacting factor 3, SV40-NLS: nuclear localization signal of the simian virus 40, VP64-AD: quadruple tandem repeat of the herpes simplex virus VP16 activation domain, PhyBNT: N-terminal version of phytochrome B, pTDH3: TDH3 promoter, Cdc1-NLS: nuclear localization sequence of yeast Cdc1, ttTDH3: TDH3 terminator, AMPr: ampicillin resistance, pSCW11: SCW11 promoter, CRE: Cre recombinase, EBD: β-estradiol-binding domain, pTIP1: TIP1 promoter, ttACS2: ACS2 terminator, pPGK1: PGK1 promoter, ttCIT1: CIT1 terminator, pZEO1: ZEO1 promoter.

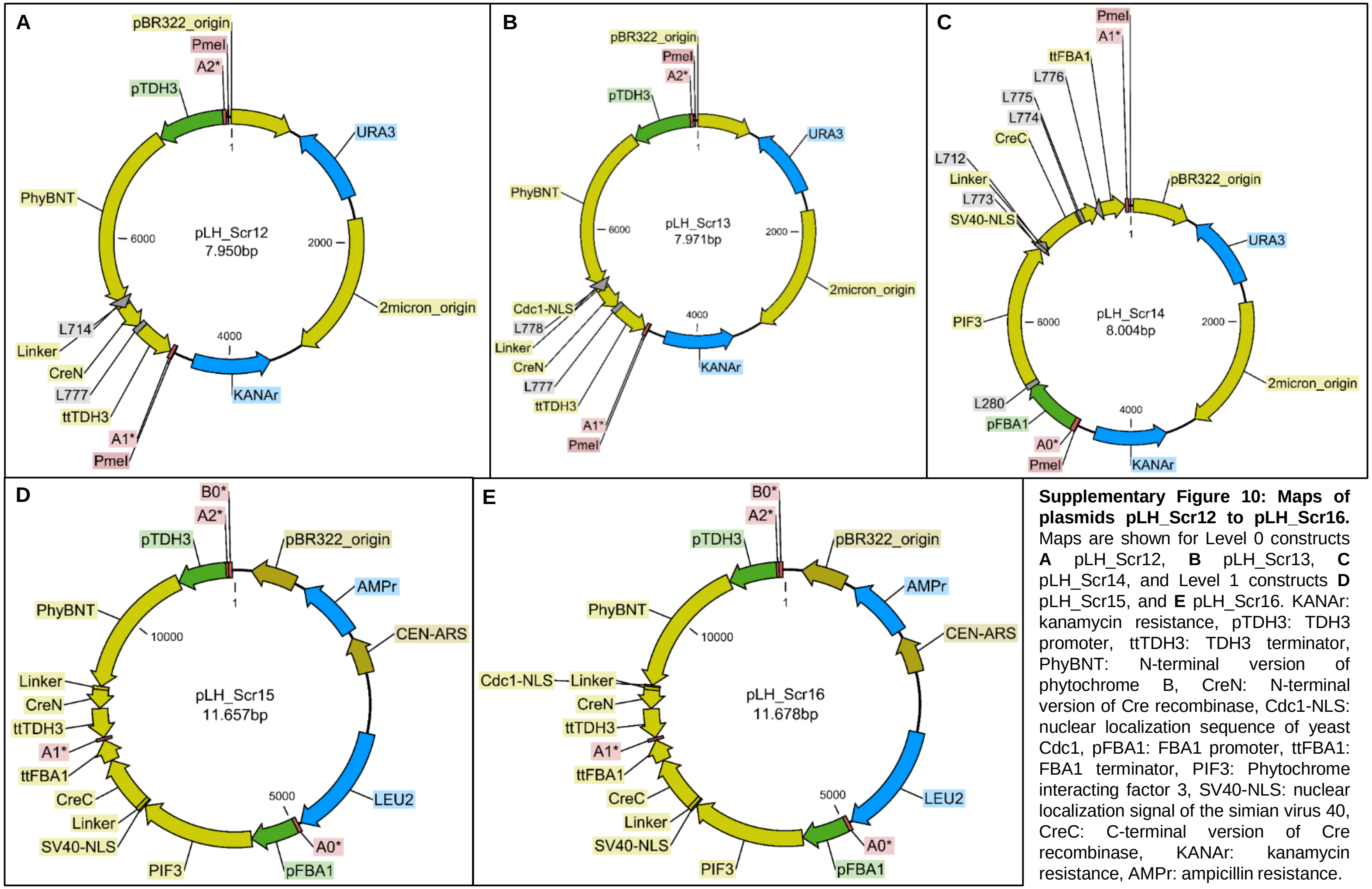

Supplementary Figure 10: Maps of plasmids pLH_Scr12 to pLH_Scr16. Maps are shown for Level 0 constructs **A** pLH_Scr12, **B** pLH_Scr13, **C** pLH_Scr14, and Level 1 constructs **D** pLH_Scr15, and **E** pLH_Scr16. KANAr: kanamycin resistance, pTDH3: TDH3 promoter, ttTDH3: TDH3 terminator, PhyBNT: N-terminal version of phytochrome B, CreN: N-terminal version of Cre recombinase, Cdc1-NLS: nuclear localization sequence of yeast Cdc1, pFBA1: FBA1 promoter, ttFBA1: FBA1 terminator, PIF3: Phytochrome interacting factor 3, SV40-NLS: nuclear localization signal of the simian virus 40, CreC: C-terminal version of Cre recombinase, KANAr: kanamycin resistance, AMPr: ampicillin resistance.

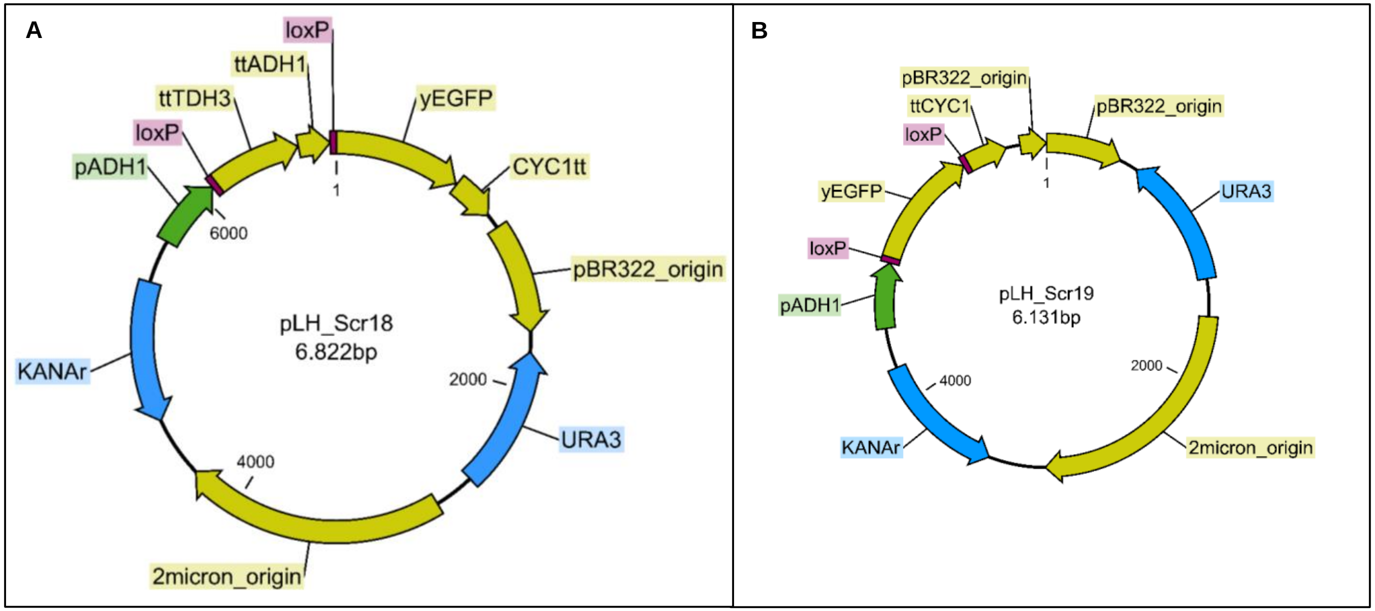

Supplementary Figure 11: Maps of plasmids pLH_Scr18 and pLH_Scr19. Maps are shown for constructs **A** pLH_Scr18 and **B** pLH_Scr19. KANAr: kanamycin resistance, pADH1: ADH1 promoter, ttTDH3: TDH3 terminator, ttADH1: ADH1 terminator, yEGFP: yeast enhanced green fluorescent protein, ttCYC1: CYC1 terminator, KANAr: kanamycin resistance.

Supplementary Table 1: List of parts with source and sequence modifications.

Assembly components	***Source***	***Modifications***	***Function***
PhyBNT	*Arabidopsis thaliana*	AA 1-621 of PhyB/ nucleotide 576 C-->A	photoreceptor
PIF3	*Arabidopsis thaliana*		interacting factor
CreC	pLM006	AA 106-343 of Cre recombinase / AA 162 V-->R / AA 170E-->K / AA 231 V-->R	C-terminal part of Cre recombinase
CreN	pLM006	AA 19-104 of Cre recombinase	N-terminal part of Cre recombinase
linker	pmCherry-CRY2-CreN		linker
SV40 NLS	oligo synthesis		nuclear localization signal
Cdc1 NLS	oligo synthesis		nuclear localization signal
FBA1 promoter	*Saccharomyces cerevisiae*		constitutive promoter
TDH3 promoter	*Saccharomyces cerevisiae*		
FBA1 terminator	*Saccharomyces cerevisiae*		terminator
TDH3 terminator	*Saccharomyces cerevisiae*		

Supplementary Table 2: List of primers used for cloning.

Name	***Sequence (5´-3´)***
L280	TCATATATAACCATAACCAAGTAATACATATTCAAAATGCCTCTGTTTGAGCTTTTC
L706	TAGATTCTGTGAAACTCTCAATAAAGGGACTTCGTCTAAGGTTTCAGTCACACTACGGATACTTTTACAACGGGAGCAGTTATTCA
L707	TGAATAACTGCTCCCGTTGTAAAAGTATCCGTAGTGTGACTGAAACCTTAGACGAAGTCCCTTTATTGAGAGTTTCACAGAATCTA
L712	CCAAGAAGAAGAGAAAGGTGgGTGGCGGTGGCTCTGGAGGTGGTGGGTCCGGAGGAGGCGGCCGCCGACCAAGTGACAGCAATGCTGTTTCACTGG
L714	TCCAGCTTATTCTGAGAGACTCTTTTAAAGAATCTGGTGGCGGTGGCTCTGGAGGTGGTGGGTCCGGAGGAGGCGGCCGCACGAGTGATGAGGTTCGCAAG
L773	GAGCCACCGCCACcCACCTTTCTCTTCTTCTTGGGGCTCG
L774	CTtAGCGCCGTAAATCAGTCGATGAGtTGCTTCAAAAATCCCTTCC
L775	ACTCATCGACTGATTTACGGCGCTAAGGATGACTCTGGTCAGAGG
L776	AAAAAACTATATCAATTAATTTGAATTAACCTAATCGCCATCTTCCAGCAGGCGCACCATTGCCCCTGTTTC
L777	TTATTTAAATGCAAGATTTAAAGTAAATTCACCTACAGCCCGGACCGACGATG
L778	CAGCTTATTCTGAGAGACTCTTTTAAAGAATCTAGGCCATTAAAGCGTAAGAAAGGTGGCGGTGGCTCTGGAGGTGGTGGGTCCGGAGGAGGCGGCCGCACGAGTGATGAGGTTCGCAAG
L802	TACATTATACGAAGTTATAGTTGATTGTATGCTTGGTATAGCTTG
L803	TACATTATACGAAGTTATATTAGTTATGTCACGCTTACATTCACGCC
L813	TATGCTATACGAAGTTATATGTCTAAAGGTGAAGAATTATTCACTGGT
L814	CCAAGCATACAATCAACTATAACTTCGTATAATGTATGCTATACGAAGTTATGTGAATTTACTTTAAATCTTGCA
L815	AAATCATAAGAAATTCGCTTAGGAATCTGTGTATATTACTGCATCT
L816	ATATACACAGATTCCTAAGCGAATTTCTTATGATTTATG
L817	TTCTTCACCTTTAGACATATAACTTCGTATAGCATACATTATACGAAGTTATGGTGTGGTCAATAAGAGC
L822	AAGCGTGACATAACTAATATAACTTCGTATAATGTATGCTATACGAAGTTATTTATTTGTACAATTCATCCATAC
L823	CCAAGCATACAATCAACTATAACTTCGTATAATGTATGCTATACGAAGTTATATGTCTAAAGGTGAAGAATTATTCACTG
