# Supplementary material for: L-SCRaMbLE as a tool for light-controlled Cre-mediated recombination in yeast
Source: Nat Commun. 2018 May 22;9:1931. doi: 10.1038/s41467-017-02208-6 (PMC5964156; doi:10.1038/s41467-017-02208-6)
Supplement: Supplementary file 3 — Description of Additional Supplementary Files [file 41467_2017_2208_MOESM3_ESM.pdf]

Description of Additional Supplementary Files

File Name: Supplementary Data 1
Description: Sequence of vector pL1A0_B0_Leu

File Name: Supplementary Data 2
Description: Sequence of vector pLH_Scr12

File Name: Supplementary Data 3
Description: Sequence of vector pLH_Scr13

File Name: Supplementary Data 4
Description: Sequence of vector pLH_Scr14

File Name: Supplementary Data 5
Description: Sequence of vector pLH_Scr15

File Name: Supplementary Data 6
Description: Sequence of vector pLH_Scr16

File Name: Supplementary Data 7
Description: Sequence of vector pLH_Scr18

File Name: Supplementary Data 8
Description: Sequence of vector pLH_Scr19

File Name: Supplementary Data 9
Description: Sequence of vector pLM006

File Name: Supplementary Data 10
Description: Sequence of vector pLM494

File Name: Supplementary Data 11
Description: Sequence of vector pRot1

File Name: Supplementary Data 12
Description: Sequence of vector pRot2

File Name: Supplementary Data 13
Description: Sequence of vector pRot4
